# Special Issue: “Respiratory Disease in the COVID-19 Era”

**DOI:** 10.3390/medicina59050886

**Published:** 2023-05-05

**Authors:** Masaki Okamoto

**Affiliations:** Department of Respirology and Clinical Research Center, National Hospital Organization Kyushu Medical Center, 1-8-1 Jigyohama, Chuo-ku, Fukuoka 810-0065, Japan; okamoto_masaki@med.kurume-u.ac.jp; Tel.: +81-92-852-0700; Fax: +81-92-847-8802

The outbreak of the viral infection known as coronavirus disease 2019 (COVID-19), caused by the novel pathogen severe acute respiratory syndrome coronavirus-2 (SARS-CoV-2), was first reported in Wuhan, China, in December 2019. Thereafter, the illness spread rapidly across the world [1]. The development of acute respiratory distress syndrome has remained the most significant risk factor for acute COVID-19-related mortality since the beginning of the outbreak [1,2,3]. In a study by Wu et al., 44 (52.4%) of 84 patients who developed acute respiratory distress syndrome attributable to COVID-19 died^2^. Aggravating factors for this condition include older age, obesity with diabetes mellitus as a complication, hypertension, and malignant disease [2,3].

COVID-19-related pneumonia exhibits specific radiological features upon high-resolution computed tomography. Li et al. found that patients with COVID-19-related pneumonia were more likely to have rounded opacities (35% vs. 17%) and interlobular septal thickening (66% vs. 43%), and were less likely to have nodules (28% vs. 71%), the tree-in-bud sign (9% vs. 40%), or pleural effusion (6% vs. 31%), when compared with patients who had influenza-related pneumonia [4].

Randomized controlled trials and observational studies of various immunosuppressive therapies have been performed in patients with COVID-19-related pneumonia. A meta-analysis suggested that corticosteroid therapy resulted in delayed virus clearance and did not improve survival or decrease the length hospital stays, the rate of admission to intensive care units, and/or the use of mechanical ventilation in patients with SARS-CoV-2, SARS-CoV, or MERS-CoV infection [5]. However, some studies have found no difference in the time to clearance of SARS-CoV-2 RNA regardless of whether corticosteroid therapy is administered [6]. The controlled open-label RECOVERY trial compared mortality between patients with COVID-19 who received oral or intravenous dexamethasone at a dosage of 6 mg, once daily, for up to 10 days and those who received standard care alone. The 28-day mortality rate (i.e., the primary outcome) was lower in patients with moderate or severe COVID-19 who received dexamethasone than in those who received standard care [7]. However, no benefit was seen in patients with mild COVID-19. In a prospective meta-analysis of 10,930 patients with COVID-19 that compared the outcomes of patients who received standard care with those of patients who received a placebo, the administration of tocilizumab and interleukin-6 antagonists was associated with lower 28-day all-cause mortality [8]. To date, the only drugs that show evidence of reducing mortality in patients with COVID-19 are corticosteroids. This finding contrasts with the results of a meta-analysis showing that corticosteroid therapy increases mortality in patients with influenza [9].

One of the chronic sequelae of COVID-19 is residual respiratory impairment after acute pneumonia. A systematic review of 13 studies that included a total of 2018 patients found that about 44.9% of COVID-19 survivors developed pulmonary fibrosis [10].

The spread of COVID-19 is abating, but is not converging. Therefore, this Special Issue presents basic and clinical research on this disease.
